# Berlin Green Framework-Based Gas Sensor for Room-Temperature and High-Selectivity Detection of Ammonia

**DOI:** 10.1007/s40820-020-00586-z

**Published:** 2021-01-25

**Authors:** Tingqiang Yang, Lingfeng Gao, Wenxuan Wang, Jianlong Kang, Guanghui Zhao, Delong Li, Wen Chen, Han Zhang

**Affiliations:** 1 Institute of Microscale Optoelectronics, Collaborative Innovation Centre for Optoelectronic Science and Technology, Key Laboratory of Optoelectronic Devices and Systems of Ministry of Education and Guangdong Province, College of Physics and Optoelectronic Engineering, Shenzhen Key Laboratory of Micro-Nano Photonic Information Technology, Guangdong Laboratory of Artificial Intelligence and Digital Economy (SZ), Shenzhen University, Shenzhen, 518060 People’s Republic of China; 2 State Key Laboratory of Advanced Technology for Materials Synthesis and Processing, School of Materials Science and Engineering, Wuhan University of Technology, Wuhan, 430070 People’s Republic of China; 3 Research Center for Materials Genome Engineering, Wuhan University of Technology, Wuhan, 430070 People’s Republic of China

**Keywords:** Berlin green framework, Gas sensor, Ammonia, Room temperature, High selectivity

## Abstract

Ammonia detection possesses great potential in atmosphere environmental protection, agriculture, industry, and rapid medical diagnosis. However, it still remains a great challenge to balance the sensitivity, selectivity, working temperature, and response/recovery speed. In this work, Berlin green (BG) framework is demonstrated as a highly promising sensing material for ammonia detection by both density functional theory simulation and experimental gas sensing investigation. Vacancy in BG framework offers abundant active sites for ammonia absorption, and the absorbed ammonia transfers sufficient electron to BG, arousing remarkable enhancement of resistance. Pristine BG framework shows remarkable response to ammonia at 50–110 °C with the highest response at 80 °C, which is jointly influenced by ammonia's absorption onto BG surface and insertion into BG lattice. The sensing performance of BG can hardly be achieved at room temperature due to its high resistance. Introduction of conductive Ti_3_CN MXene overcomes the high resistance of pure BG framework, and the simply prepared BG/Ti_3_CN mixture shows high selectivity to ammonia at room temperature with satisfying response/recovery speed.

**Supplementary Information:**

The online version of this article (10.1007/s40820-020-00586-z) contains supplementary material, which is available to authorized users.

## Highlights

- Berlin green (BG) framework is highly promising for ammonia detection demonstrated by both theoretical and experimental investigations.
- BG/Ti_3_CN mixture shows high selectivity to ammonia at room temperature with satisfying response/recovery speed.

**Supplementary Information:**

The online version of this article (10.1007/s40820-020-00586-z) contains supplementary material, which is available to authorized users.

## Graphical abstract

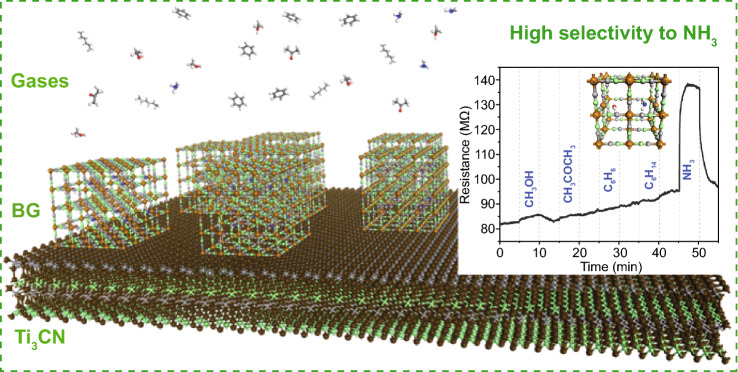

## Introduction

Ammonia is always considered as a big frontier in public health protection not only due to its malodor but also because of its ability to combine with NO_*x*_ or SO_*x*_ into NH_4_^+^ salt, a main component of PM2.5 [1]. Human's agricultural activity, use of fertilizers and animal manure, is the most significant source of ammonia emitted to atmosphere [2]. The gas is also extensively generated in industrial sectors, such as fertilizer production, chemical engineering, and food processing [2]. Concentration of human exhaled ammonia can be a sign for medical diagnosis, and abnormal increase in the concentration may originate from ulcers caused by helicobacter pylori, hepatic encephalopathy, kidney disorder, or bacterial in oral cavity [3]. Developing a high-quality ammonia gas sensor possesses great potential in atmosphere environmental protection, agriculture, industry, and rapid medical diagnosis, etc. [4].

There are abundant categories of gas sensors, among which chemiresistive type has been drawing extensive attention on account of its simplicity in sensor structures, maturity in micro- or nano-device fabrication, and compatibility with modern electronics as well as low cost [5–7]. The key part of chemiresistive gas sensor is the sensing materials, generally including metal oxides semiconductors (MOSs), conductive polymers, quantum dots, and so on [8–12]. For those sensing materials, it still remains a big challenge to balance the sensitivity, selectivity, working temperature and response/recovery speed.

Metal organic frameworks (MOFs) are a class of materials, whose crystalline is in framework structure constructed by connecting metal center with organic linker [13–16]. Such structure offers ultrahigh specific surface areas, regular and tunable pores, and open metal sites to molecules, rendering MOFs very attractive for improving gas sensing performance [17–22]. Campbell et al. have reported that conductive 2D MOFs can be utilized as active materials for detecting volatile organic compounds (VOCs) and a sensor array based on MOFs with well-designed structure is able to discriminate different VOCs [17]. MOFs have been demonstrated to be capable of improving selectivity as a coating on MOSs nanostructures. Drobek et al. have coated a thin ZIF-8 (a kind of zeolitic imidazolate framework, 2-methylimidazole zinc salt) film on ZnO nanowires, and the nanocomposite exhibits remarkable selectivity to hydrogen gas compared with pristine ZnO nanowires [18]. Yao et al. have deposited ZIF-CoZn (an analogue of ZIF-8) on ZnO nanowire array to fabricate a sensor showing better performance to acetone with interference of humidity [19]. Additionally, a Janus nanostructure of Au@ZnO@ ZIF-8 has been prepared and shows fast response to formaldehyde at room temperature [20]. Li et al. have prepared various kinds of MOFs which show outstanding gas performance to formic acid as well as ammonia and ammines [23–25]. Specially, a 2D copper–organic framework and a 3D cadmium–organic framework both show perfect selectivity to ammonia [24, 25], whereas the identification is based on impedance spectroscopy which is not suitable for device minimization. To the best of our knowledge, there are few reports of MOF-based chemiresistive sensor able to selectively detect ammonia at room temperature. Although a heterostructured MOF-on-MOF film shows response to ammonia at room temperature, its drawback is cross-sensitivity to benzene [21].

**Fig. 1:**
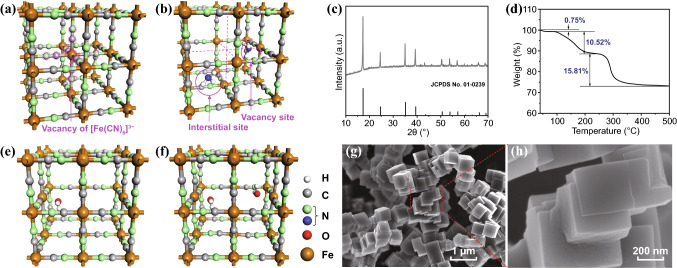
**a** Atomic structure of BG, **b** absorbed ammonia molecule at two sites in BG lattice, **c** XRD patterns of BG powder, **d** TGA pattern of the prepared BG powder, **e**, **f** atomic structures of Fe[Fe(CN)_6_]_0.75_·*x*H_2_O (*x* = 1, 2), and **g**, **h** low- and high-resolution FESEM images of BG framework

Prussian blue (PB, KFe^III^Fe^II^(CN)_6_) and its analogues (A_i_M_j_[M′(CN)_6_]_k_, A is an alkaline metal, and M and M′ transition metals), classified as MOFs with transition metal as center atom and –CN– as linker, have raised growing interest in molecular magnets, energy storage and molecule absorbents [26–30]. Berlin green (BG, Fe^III^Fe^III^(CN)_6_) is one analogue of PB, whose framework structure is shown in Fig. 1a, with Fe^III^ being the center and –CN– the linker. [Fe(CN)_6_]^3−^ vacancy is unavoidably introduced in BG framework by conventional aqueous method [31, 32]. The vacancy is harmful for the role as capacity cathodes for alkali ion batteries, whereas it offers abundant sites for absorbing molecules and renders Fe^III^ atom exposed to molecules [31, 32]. Figure 1b shows two kinds of sites for ammonia absorption provided by the vacancy-contained structure: one is the vacancy site where ammonia molecule can coordinate with face-centered Fe^III^ atom, and the other is the interstitial site which is a confined space surrounded by a cubic framework [28]. Therefore, it has been discovered that BG framework exhibits extraordinary performance in capturing ammonia molecule [28]. The captured ammonia molecule may have impacts on the conductivity of the BG framework; thus, BG framework can be a sensing material to detect ammonia.

The low conductivity of BG makes it difficult to collect electrical signal, which can be overcame by introducing conductive component. 2D materials with high specific surface and high conductivity have been extensively utilized as backbones to support and disperse sensing materials as well as to transport electrical signal [11, 33]. The backbone should also be capable of interacting with BG framework to form intimate contact and avoid separation of two components. Transition-metal carbides/carbonitrides, well known as MXenes, are a new member of 2D material family and have been applied to various sensors due to their tunable interplanar space as well as excellent conductivity [34–38]. Additionally, their abundant surface terminations, such as OH, O and F, are endowed with a lone-paired electrons, rendering MXenes have high tendency to coordinate with Fe atoms of BG framework. Lee et al. have reported that a MXene of Ti_3_C_2_ shows response to ammonia, methanol, ethanol and acetone at room temperature, while the selectivity is poor [34]. Nanohybrids of Ti_3_C_2_/WSe_2_ have also been demonstrated to be able to selectively detect VOCs at room temperature [36]. In contrast to Ti_3_C_2_, Ti_3_CN, whose structure and conductivity are comparable to its analogue [39], is seldom utilized in gas sensor application. It has been revealed that relatively more electronegative nitrogen compared to carbon at the MXene's X sites gives stronger bonding to the electropositive M, and theoretical investigations have proved higher electronic conductivity and more catalytically active surface on nitride MXenes [40].

Herein, BG framework is prepared by a facile aqueous method. Based on material characterization, atomic structure of BG framework is established, and a density functional theory (DFT) simulation is subsequently executed to evaluate feasibility of BG framework to detect ammonia gas. Experimentally, pure BG framework shows remarkable response to ammonia among the range of 50–110 °C. Introduction of Ti_3_CN overcomes high resistance of BG framework, and the prepared BG/Ti_3_CN mixture exhibits high selectivity to ammonia at room temperature with satisfying response and recovery speed.

## Experimental and Computational Details

### Materials

Ti_3_AlCN (~ 600 mesh) was purchased from Forsman Scientific Co., Ltd. Hydrofluoric aqueous solution (HF, 40%) and ethanol (99.9%) were purchased from Macklin Inc. K_3_Fe(CN)_6_ (99.5%) and HCl (36–38%) were purchased from Sinopharm Chemical Reagent Co. Ltd. Aqueous ammonia (25–28%), methanol (99.9%), acetone (99.9%), benzene (99.9%) and n-hexane (99.9%) to generate gases for sensing test were also bought from Sinopharm Chemical Reagent Co. Ltd. All these chemical reagents were used as received, and deionized (DI) water was utilized in the whole synthetic process.

### Preparation of BG

BG powders were synthesized by a facile hydrolytic precipitation. Specifically, a certain amount of K_3_Fe(CN)_6_ was dissolved in 0.1 mol L^−1^ HCl aqueous solution with molar ratio of 1/3; then, the mixture was vigorously stirred at 80 °C for 12 h. Afterward, a dark green product was precipitated, which was further collected by suction filtration and several-time wash with deionized water. Finally, the product was dried at 60 °C at vacuum condition to get dark green powder.

### Preparation of Multilayer Ti_3_CN MXene

10 g Ti_3_AlCN powder was immersed in 60 mL 40% HF aqueous solution for 48 h at room temperature. After etching procedure, superfluous HF was removed by repeatedly washing with DI water and centrifuging at 3000 rpm until the pH reached ~ 6. The portion of MXene suspension was collected by filtration through a cellulose membrane (40 mm diameter with pore size of 0.2 μm) and washed with DI water and ethanol for several times. The obtained light brown powder was the multilayer Ti_3_CN MXene, which was dried in vacuum at 60 ºC for 24 h and stored under the protection of argon.

### Preparation of BG/Ti_3_CN Mixture

The as-synthesized multilayer Ti_3_CN MXene was readily mixed with BG powder in ethanol at various weight ratios (1:1, 2:1, 4:1, and 6:1). Taking the preparation BG/Ti_3_CN mixture with weight ratio of 1:1 (BG/Ti_3_CN (1:1)) as an example, 10 mg Ti_3_CN (weighted by balance) was dispersed in 100 μL ethanol in a one-milliliter vial and sonicated for 1 min, and 10 mg BG powder was then added in the vial and sonicated for another 1 min. Similar procedure was used for the preparation of BG/Ti_3_CN mixtures with various ratios.

### Materials Characterization

The crystal structures and microstructures of the as-prepared samples were characterized by X-ray diffraction (Pert-Pro, PANalytical, Netherlands). Thermal gravimetric analysis (TGA) was executed to confirm the water content by using thermal analyzer (STA409PC, NETZSCH) in the N_2_ atmosphere with a heating rate of 5 °C min^−1^. The micro-morphologies of BG and BG/Ti_3_CN were revealed by field-emission scanning electron microscopy (FESEM) (S-4800, Hitachi, Japan), and an energy dispersive spectrometer (EDS) mapping was performed to analyzed the element distribution of BG/Ti_3_CN composite. The Raman spectra were recorded from a LabRam HR Evolution (Horiba) Raman spectrometer using the second harmonic (*λ* = 532 nm) of a pulsed Nd:YAG laser.

### Fabrication of Gas Sensor Devices and Measurement of Gas Sensing Properties

Gel of BG or BG/Ti_3_CN, which was simply prepared by adding a suitable amount of ethanol to the sensing material and milling it for several minutes, was painted on a ceramic substrate to form a uniform film bridging two parallel gold electrodes. The substrate painted with sensing materials was then dried in vacuum at 80 °C to remove the ethanol molecules.

Gas sensing properties were measured by a commercial gas sensing measurement system (WS-30A, Zhengzhou Winsen Technology Corp., China), whose schematic diagram is shown as Scheme S1. A certain load resistance is in series circuit of the gas sensor under an overall voltage, and the collected signal is the output voltage of load resistance. The resistance variation of sensor device can be obtained according to Eq. (1):

1 $$\frac{{R_{{{\text{Sensor}}}} }}{{R_{{{\text{Load}}}} }} = \frac{{U_{{{\text{Overall}}}} - U_{{{\text{Output}}}} }}{{U_{{{\text{Output}}}} }}$$

The sensor device can be heated by a heating wire which is underneath the sensing materials, and a certain temperature can be reached by applying a specific heating voltage. The gas sensing test was a kind of combination of static and dynamic method, and the details are illustrated in Fig. S1. The gas response is defined as the ratio of resistance in testing gas and that in air, i.e., *R*_gas_/*R*_air_.

### Computational Details

Periodic density functional calculations were performed by using Dmol3 4.4 program, and general gradient approximation (GGA) in the form of a Perdew–Burke–Ernzerhof (PBE) was selected for the exchange–correlation functional [41–43]. The van der Waals interaction was taken into consideration by using TS method for DFT-D correction. The double numeric quality basis set with polarization functions (DNP) was used. The inner electrons of Fe atoms were kept frozen and replaced by an effective core potential (ECP), and other atoms were treated with an all-electron calculation. Brillouin-zone integrations were performed using a 3 × 3 × 3 and 3 × 3 × 1 Monkhorst–Pack grid for the primitive BG cell and surface supercell, respectively. The tolerances of energy, force, and displacement convergence for geometry optimization were 2 × 10^−5^ hartree, 4 × 10^−3^ hartree Å^−1^, and 5 × 10^−3^ Å, respectively. Several possible configurations of ammonia on BG surface of in BG lattice were established for optimization, and all of the atomic structures presented in this paper were the most stable configurations.

## Results and Discussion

### Characterization and Atom Structure of BG Framework

Figure 1c shows the XRD patterns of BG framework. All diffractive peaks can be assigned to the cubic iron cyanide (JCPDS No. 01-0239) with chemical formula of Fe[Fe(CN)_6_]_0.75_, suggesting that the cubic cell volume is 10.2 × 10.2 × 10.2 Å^3^ and there is about 25% of [Fe(CN)_6_]^3−^ vacancy in the BG framework. Because of the high percentage of vacancy, water molecule is hardly avoidable to embed into BG lattice during preparation procedure in aqueous solution. TGA under nitrogen atmosphere demonstrates 0.75 wt% weight loss when temperature is elevated from 40 to 100 °C (Fig. 1d), suggesting desorption of water molecule absorbed on surface. Additionally, there is 10.52 wt% weight loss when temperature is further elevated from 100 to 225 °C, suggesting desorption of lattice water molecule. The higher temperature can result in severer weight loss, which is attributed to the decomposition of BG framework. The 10.52 wt% weight loss of lattice water demonstrates that there are about 1.3 H_2_O molecules on average embedded in each Fe[Fe(CN)_6_]_0.75_ primitive cell. Based on these characterizations, atomic structure of BG is established, as shown in Fig. 1e, f. Some Fe[Fe(CN)_6_]_0.75_ lattices are embedded with just one H_2_O molecule, while some others two. The only one H_2_O molecule tends to embed at the vacancy site with oxygen atom coordinating with face-centered Fe atom. When there are two lattice water molecules, they are more energetically stable to coordinate with Fe atom face to face. Figure 1g, h shows the FESEM image of the prepared BG powder. The powder is microcube shape with smooth and clean surface, and mostly the microcube grows from another one forming building block-like structure.

### DFT Simulation of Ammonia Absorption and Insertion

**Fig. 2:**
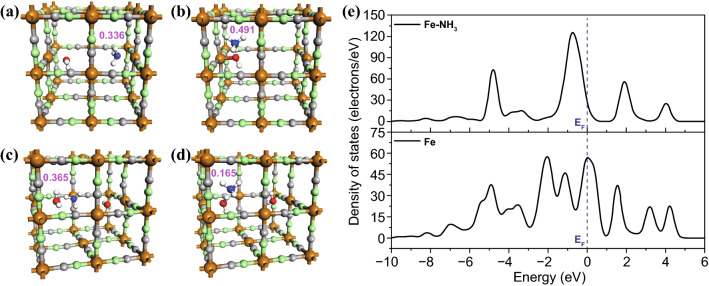
Atomic structures of ammonia molecule inserted into BG lattice: **a**, **b** vacancy site and interstitial site for Fe[Fe(CN)_6_]_0.75_·H_2_O, and **c, d** vacancy site and interstitial site for Fe[Fe(CN)_6_]_0.75_·2H_2_O (the number in red is the total Mulliken Charge of absorbed ammonia molecule); **e** DOS patterns of 3d orbital of face-centered Fe atom before and after ammonia absorption of panel **a**

It has been demonstrated that ammonia molecule can be absorbed at vacancy site or interstitial site [28]. Herein, atom configuration of ammonia molecule in BG framework is simulated through DFT calculation according to the established BG lattice. Ammonia molecule is initially put around the two particular sites in BG lattice embedded with one or two lattice water molecules, and further optimization is performed to find the most stable configuration. Figure 2a shows optimized configuration of ammonia molecule at vacancy site which is face to face with the H_2_O molecule in Fe[Fe(CN)_6_]_0.75_·H_2_O lattice, and the nitrogen atom of NH_3_ coordinates with face-centered Fe atom. Figure 2b shows the optimized configuration of ammonia molecule at interstitial site besides the H_2_O molecule, and ammonia molecule spontaneously grabs a hydrogen atom from H_2_O to produce NH_4_^+^ and hydroxyl. The reaction has been demonstrated by infrared (IR) spectra [28], and the animation of the reaction can be found in Video S1. Absorption energy of ammonia molecule in Fe[Fe(CN)_6_]_0.75_·H_2_O is − 211.2 and − 173.5 kJ mol^−1^, respectively, at the vacancy site and interstitial site, suggesting a little higher tendency of ammonia molecule at vacancy site. Figure 2c, d shows the ammonia molecules at vacancy and interstitial sites in Fe[Fe(CN)_6_]_0.75_·2H_2_O lattice, and their absorption energy is calculated to be − 182.7 and − 146.22 kJ mol^−1^, respectively. The absorption also causes charge transfer from ammonia to BG framework, which is demonstrated by Mulliken Charge of ammonia molecule. According to DFT calculation, Mulliken Charges of NH_3_ are positive whether the molecule is at vacancy site or interstitial site in Fe[Fe(CN)_6_]_0.75_·*x*H_2_O (*x* = 1, 2) lattice. The largest value of Mulliken Charge is 0.491 when the molecule is at interstitial site in Fe[Fe(CN)_6_]_0.75_·H_2_O lattice, and the smallest is 0.165 when the molecule is at interstitial site in Fe[Fe(CN)_6_]_0.75_·2H_2_O lattice. Positive Mulliken Charge suggests that NH_3_ molecule injects electron into BG lattice. Density of states (DOSs) of 3d orbital of face-centered Fe atom in Fig. 2a is calculated before and after the ammonia absorption, as shown in Fig. 2e. For uncoordinated Fe atom, there is a DOS peak at Fermi level (*E*_F_), suggesting that a large part of 3d orbital is not occupied. Pajerowski et al. have theoretically and experimentally demonstrated that electron conduction mechanism in BG around room temperature is attributed to electron’s hopping onto nearest-neighbor unoccupied state [44]. Simply speaking, BG can be considered as a p-type semiconductor, and the unoccupied state around Fermi level is beneficial for BG’s conductivity. According to DFT calculation, DOS at *E*_F_ is much lower after ammonia’s absorption and there is a large gap above E_F_, suggesting there is much fewer unoccupied state for electron to hopping onto. Therefore, computational results demonstrate that BG is highly likely to possess gas response to ammonia, and BG must show enhancement in resistance upon exposing to ammonia.

### Gas Sensing Performance and Mechanism of BG Framework

**Fig. 3:**
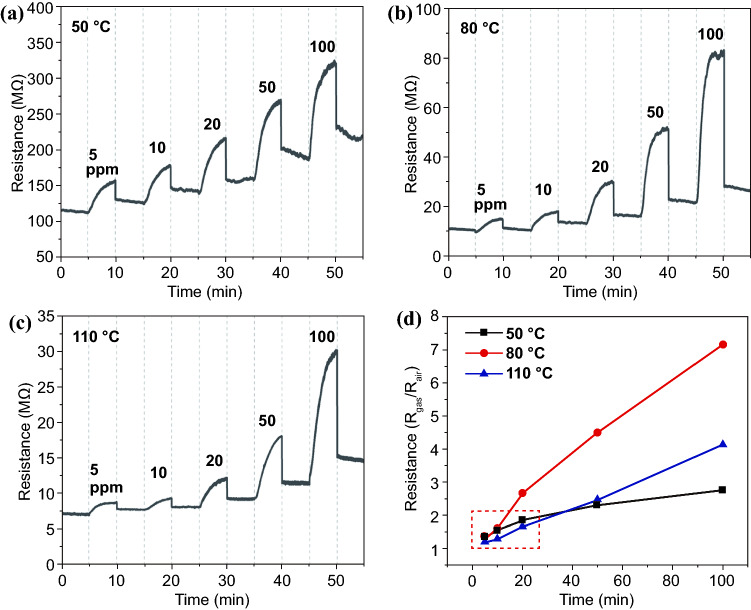
Resistance variation of BG sensor to different concentrations of ammonia at **a** 50 °C, **b** 80 °C, and **c** 110 °C and **d** correlation between gas response and ammonia concentration at different temperatures

Figure 3a–c shows the gas response of BG framework to ammonia at different temperatures. It is clear that the BG framework shows remarkable response to ppm-level ammonia at 50, 80, and 110 °C. Resistance of BG sensor increases when ammonia gas is injected, which is consistent with computational results. Ammonia is one typical reducing gas, which donates electron to p-type semi-conductive BG framework upon being absorbed. The electron from ammonia will fill up the unoccupied state around Fermi level of BG framework, making it less possible for electron below Fermi level to hopping onto nearest-neighbor state. Improving ammonia concentration definitely resulted in more enhanced resistance. Because of high affinity of ammonia and accumulation of BG crystals, the resistance continuously increases even at the end of five-minute response process after injecting every specific concentration of ammonia, suggesting slow response speed. Recovery property of BG framework is also not satisfying. Figure S1 clearly shows detailed process for testing recovery property, and there are the five-minute exposure to air and another five-minute interval to stabilize temperature and gas flow before collecting resistance signal. In spite of that, the resistance still cannot recover to original level.

Figure 3d compares the correlation between gas response and ammonia concentration at different temperatures. Apparently, the response increases with the improvement of ammonia concentration, while there is a little difference for the increase trend. At 50 °C, increase rate of response is gradually deteriorated with improving the concentration. In contrast, the increase rates are nearly constants at 80 and 110 °C, with the former being evidently larger. In detail, the responses to 5 and 10 ppm ammonia at 50 °C is comparable to those at 80 °C, and is higher than those at 110 °C. For the response to 20 ppm, the one at 80 °C is the largest, followed by those at 50 and 110 °C. When the concentration is further improved, the response at 110 °C exceeds that at 50 °C but far lower compared with that at 80 °C. This characteristic may be affected by the relative ability of ammonia’s absorption onto BG surface and insertion into BG lattice.

**Fig. 4:**
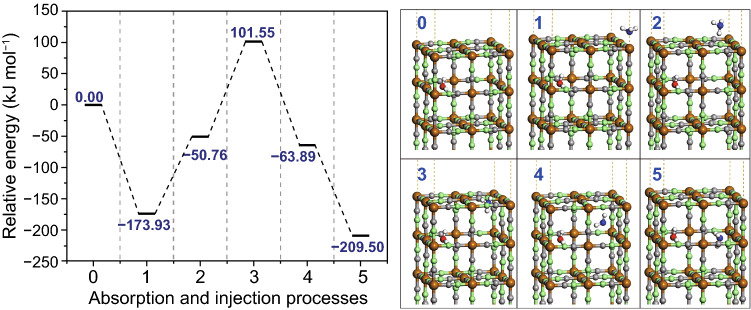
Absorption and insertion processes of ammonia molecule onto surface and into lattice of BG framework

It is simulated that the ammonia molecule’s absorption onto the surface and insertion into the lattice of BG generally takes five procedures (Fig. 4). Firstly, ammonia molecule has high tendency to be absorbed onto BG surface, with N atom downward to coordinate with surface Fe atom bonded to five N atoms, and the process is highly exothermic. Secondly, the molecule migrates to the interstitial site at surface with H atom downward. Thirdly, the molecule penetrates through the interstice. When the N atom is at the same plane with surface atoms, relative energy of the whole system reaches the peak. Fourthly, the molecule is successfully inserted at interstitial site but still in a metastable configuration. Finally, the molecule moves to vacancy site to reach a more stable condition. The simulation suggests that absorption process and the whole insertion process are exothermic, while there is a high energy barrier for ammonia at surface to insert into lattice.

Based on the simulation, the correlation between response and ammonia concentration at different temperatures can be explained. Relative low temperature, i.e., 50 °C, is more favorable for ammonia absorption [45], while the ammonia insertion is some restricted. Low concentration of ammonia can mostly be absorbed onto the surface, but much fewer can be inserted into the lattice. The ammonia on the surface is also able to arouse charge transfer and resistance enhancement. However, with improving ammonia concentration, not all of the ammonia molecule can be absorbed owing to definite surface area, and the insertion process is also restricted. As a consequence, the response to high-concentration ammonia is somewhat suppressed. At 80 °C, even though the absorption is slightly suppressed, the insertion is more facilitated. Accordingly, the response to low-concentration ammonia at 80 °C is comparable with that at 50 °C, and the response to more concentrated ammonia is much higher at 80 °C. At 110 °C, the absorption is significantly weakened, resulting in much fewer ammonia molecules absorbed on the surface. The amount of ammonia molecule inserted into the lattice is also reduced due to the fewer molecules on the surface. Hence, the lowest response is observed at 110 °C to low-concentration ammonia. For high concentration of ammonia, the response at 110 °C exceeds that at 50 °C, since the high concentration of ammonia provides chance for more molecules to insert into BG lattice. However, the amount of inserted molecule at 110 °C is much fewer than that at 80 °C.

Influence of humidity is also investigated on the response of pure BG framework to ammonia. As shown in Fig. S2, response to 20 ppm ammonia increases with improving humidity at working temperature of 80 °C. The underlying reason is mainly that NH_3_ molecule has hydrogen bond interaction with H_2_O molecule, and they can react and transform into NH_3_·H_2_O or even NH_4_^+^ and hydroxyl in BG lattice, as is demonstrated by our DFT simulation. These reactions can even induce severer charge transfer. Accordingly, ambient humidity is beneficial for the response of BG to ammonia.

### Characterization and Gas Sensing Performance of BG/Ti_3_CN Mixture

Based on above results, it is reasonable to believe that BG may show response to ammonia at room temperature because ammonia absorption is feasible. Room-temperature detection will simplify the configuration of sensor device by removing redundant heating circuit [46]. However, the resistance of pure BG framework is too high to collect electrical signal at room temperature. To overcome the dilemma, Ti_3_CN, one kind of MXene materials with accordion-like structure, perfect conductivity and abundant surface terminations, is simply mixed with BG to reduce the resistance. Figure S3 shows resistance variation of BG/Ti_3_CN mixture by different ratios, and it indicates that the resistance regularly decreases with increment in Ti_3_CN content mainly due to the perfect dispersion of two components. When the weight ratio of MXene is 50%, i.e., BG/Ti_3_CN (1:1), the resistance is at the level of 100 million Ohms, which can be detected by the testing system.

**Fig. 5:**
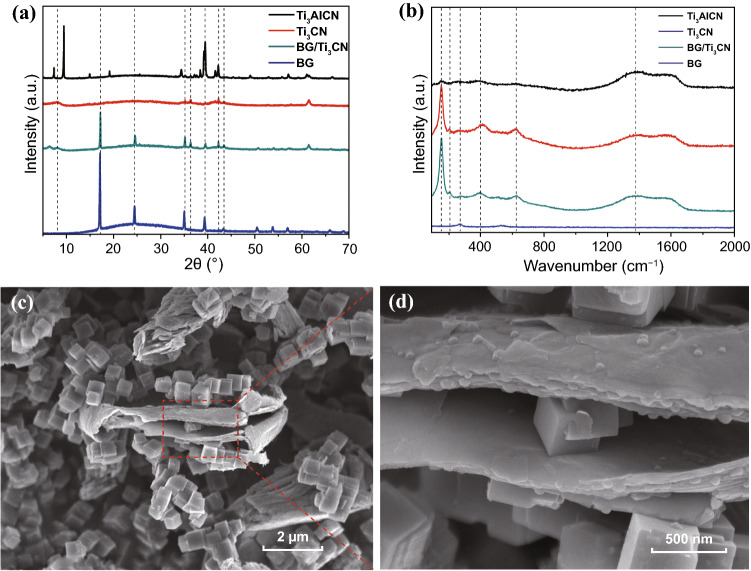
**a** XRD patterns and **b** Raman spectra of Ti_3_AlCN, Ti_3_CN, BG, and BG/Ti_3_CN (1:1). **c, d** FESEM images in a different amplification of BG/Ti_3_CN BG/Ti_3_CN (1:1)

Figure 5a shows XRD patterns of Ti_3_AlCN, Ti_3_CN, BG, and BG/Ti_3_CN (1:1) mixture. After Ti_3_AlCN is etched, peak (2θ) at 9.6 degree is much reduced and the peak shifts to lower position, suggesting destruction of the layered structure and expansion of inter-layer distance. The peaks in XRD pattern of BG/Ti_3_CN mixture can be well assigned to BG and Ti_3_CN, respectively. Figure 5b shows Raman spectra of Ti_3_AlCN, Ti_3_CN, BG, and BG/Ti_3_CN (1:1) mixture. It sees that a peak around 155 cm^−1^ is much strengthened after Ti_3_AlCN is etched into Ti_3_CN, and two other peaks around 410 and 620 cm^−1^ are remained. Two broad peaks between 1200 and 1800 cm^−1^ are the characteristics for the D- and G-modes of graphitic carbon: the former D-band generally relates to the disordered graphite formed by the defects in carbon-based materials and the latter G-band represents the stacking of the graphite hexagon network plane [39]. Even though the peak of BG is not evident in Raman spectrum, it is sufficient to demonstrate the coexistence of BG and Ti_3_CN based on the aforementioned XRD and Raman results. Figure 5c, d demonstrates FESEM images of BG/Ti_3_CN (1:1) mixture. It can be seen that BG powder maintains morphology of microcubes and Ti_3_CN is in morphology of microsheets. The microcubes and microsheets are evenly dispersed, with some of microcubes deposited on Ti_3_CN surface and some sandwiched by Ti_3_CN microsheets. Figure S4 shows EDS mapping of BG/Ti_3_CN (1:1). It is clear that C, N, Ti, Fe elements are evenly distributed, indicating BG and Ti_3_CN are well mixed.

**Fig. 6:**
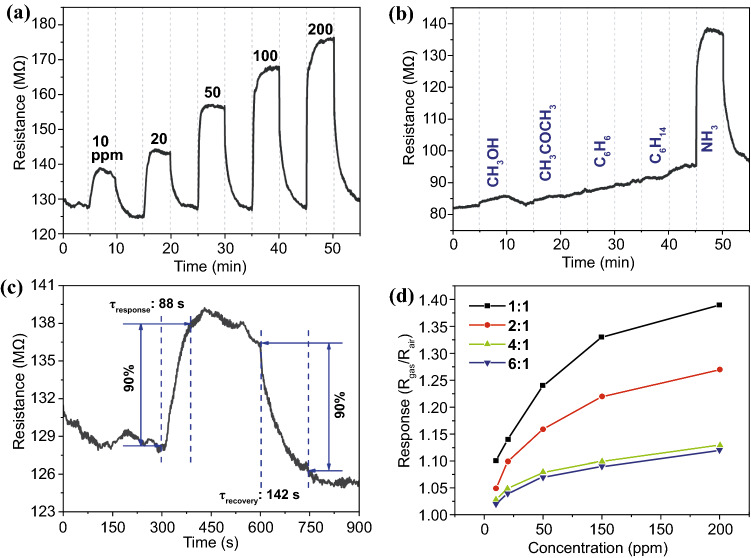
Gas sensing performance at room temperature: **a** resistance variation of BG/Ti_3_CN (1:1) sensor to different concentrations of ammonia, **b** resistance variation of BG/Ti_3_CN (1:1) sensor to different gases of 100 ppm, **c** response and recovery time of BG/Ti_3_CN (1:1) to 10 ppm ammonia, and **d** correlation between gas response and ammonia concentration of BG/Ti_3_CN by different ratios

Gas sensing properties of BG/Ti_3_CN mixture are further tested at room temperature (Fig. 6). BG/Ti_3_CN (1:1) shows remarkable response to different concentrations of ammonia with the resistance immediately jumping up after the injection of ammonia. The resistance severely increases with improving the concentration. Additionally, the resistance quickly recovers to original value when BG/Ti_3_CN (1:1) is re-exposed to air, suggesting good recovery and reproducibility of the sensor device. Furthermore, the sensor shows high selectivity to ammonia compared to methanol, acetone, benzene, and n-hexane. As shown in Fig. 6b, the resistance of BG/Ti_3_CN (1:1) increases slightly upon injection of methanol, while there is almost no change in resistance when acetone, benzene and n-hexane are injected. However, injection of ammonia leads to remarkable enhancement of resistance. Response and recovery properties are also tested, and the measurement details are illustrated in Fig. S1c, d. Response and recovery time to 10 ppm ammonia is 88 and 142 s, respectively (Fig. 6c), which is superior to that of pristine BG as well as MOF-on-MOF film [21]. Although ammonia identification can be realized by various sensing materials, as listed in Table 1, the BG/Ti_3_CN mixture in this work still possess superiorities in term of overall performance, including response at room temperature, selectivity as well as response/recovery speed.

**Table 1:** Ammonia sensing performance of chemiresistive sensor based on different materials

Sensing materials	Gas response (*R*_air_/*R*_gas_ or *R*_gas_/*R*_air_) to ammonia (ppm)	Selectivity	Working temperature	Response/recovery time
CeO_2_ [47]	6.08 (to 80)	Excellent	25 °C	~ 750 s/2750 s
Co_3_O_4_ [48]	~ 2 (to 10)	Good	RT	~ 450 s/1000 s
PbS QDs/rGO [11]	1.43 (to 750 ppb)	Unsatisfied	RT	None
rGO/Co_3_O_4_ [49]	1.54 (to 50)	Good	20 °C	4 s/5 min
ZnO/CNT [50]	6.4 (to 100)	Good	RT	20 s/420 s
MOF-on-MOF film [21]	~ 3 (to 100)	Unsatisfied	RT	~ 80 s/ ~ 642 s
BG/Ti_3_CN (This work)	1.33 (to 100)	Excellent	RT	88 s/142 s

QDs, rGO, and CNT represent quantum dots, reduced graphene oxide, and carbon nanotube, respectively. RT means room temperature

Gas sensing performances of BG/Ti_3_CN mixture by different ratios are also tested at room temperature with the results shown in Figs. 6d and S3. Even though the increment in Ti_3_CN content results in lower response, the response increases with improving ammonia concentration, which is independent of Ti_3_CN content. In addition, the increase trend shown in Fig. 6d suggests that increase rate degrades with improving ammonia concentration, which is similar to the trend of BG framework’s response at 50 °C. According to above explanation, the response to ammonia at room temperature should be mainly attributed to the absorption process at BG surface.

**Fig. 7:**
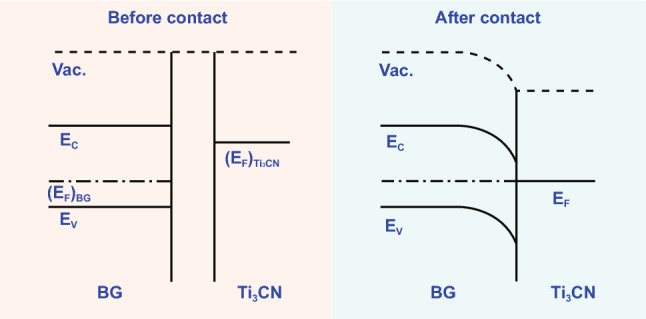
Schematic of energy band structure at interface between Ti_3_CN and BG before and after contact

For the conduction mechanism across the interface between BG and Ti_3_CN, as shown in Fig. 7, the Fermi level of p-type BG must locate at a deep level [44], and the Fermi level of Ti_3_CN is at relatively shallow level similar to that of Ti_3_C_2_ [36]. Once the two components contact, electron definitely flows from Ti_3_CN to BG until the Fermi level of BG is raised up to that of Ti_3_CN, resulting in a typical Ohmic contact [5]. The Ohmic contact between BG and conductors has also been experimentally demonstrated [44]. When the BG/Ti_3_CN composite is exposed to NH_3_, NH_3_ molecules are more prone to be attracted by BG framework. Because of ammonia’s ability to donate electron, the conductivity of p-type BG will be depressed. For the Ohmic-contacted mixture, the whole conductivity will be definitely depressed and thus the resistance will be enhanced.

## Conclusions

In summary, both DFT simulation and experimental investigation demonstrate that BG is a highly promising sensing material for ammonia detection. Vacancy in BG lattice offers abundant sites for ammonia absorption, and the absorbed ammonia transfers sufficient electron to BG to arouse remarkable enhancement in resistance. Pure BG shows response to ammonia at temperature region of 50–110 °C with the highest response at 80 °C, which is jointly influenced by ammonia’s absorption onto BG surface and insertion into BG lattice. Introduction of conductive Ti_3_CN MXene overcomes the high resistance of pure BG framework at room temperature. The simply prepared BG/Ti_3_CN mixture shows high selectivity to ammonia at room temperature with satisfying response and recovery speed.

## Supplementary Information

Below is the link to the electronic supplementary material.

Supplementary Information1 (DOCX 1591 kb)

Supplementary Information1 (AVI 1647 kb)
