# Supplementary material for: Berlin Green Framework-Based Gas Sensor for Room-Temperature and High-Selectivity Detection of Ammonia
Source: Nanomicro Lett. 2021 Jan 25;13:63. doi: 10.1007/s40820-020-00586-z (PMC8187535; doi:10.1007/s40820-020-00586-z)
Supplement: Supplementary file 1 — Supplementary Information1 (DOCX 1591 kb) [file 40820_2020_586_MOESM1_ESM.docx]

**Electronical Supporting Materials**

**Berlin Green Framework-Based Gas Sensor for Room-Temperature and High-Selectivity Detection of Ammonia**

**Tingqiang Yang,^a,#^ Lingfeng Gao,^a,#^ Wenxuan Wang,^b,#^ Jianlong Kang,^a^ Guanghui Zhao,^c^ Delong Li,^a,^* Wen Chen,^b,^* Han Zhang^a,^***

^a^Institute of Microscale Optoelectronics, Collaborative Innovation Centre for Optoelectronic Science & Technology, Key Laboratory of Optoelectronic Devices and Systems of Ministry of Education and Guangdong Province, College of Physics and Optoelectronic Engineering, Shenzhen Key Laboratory of Micro-Nano Photonic Information Technology, Guangdong Laboratory of Artificial Intelligence and Digital Economy (SZ), Shenzhen University, Shenzhen, 518060, P. R. China

^b^State Key Laboratory of Advanced Technology for Materials Synthesis and Processing, School of Materials Science and Engineering, Wuhan University of Technology, Wuhan, 430070, P. R. China

^c^Research Center for Materials Genome Engineering, Wuhan University of Technology, Wuhan, 430070, P. R. China

[*] Correspondents:

E-mail: [hzhang@szu.edu.cn](mailto:hzhang@szu.edu.cn) (Prof. Han Zhang)

E-mail: [chenw@whut.edu.cn](mailto:chenw@whut.edu.cn) (Prof. Wen Chen)

E-mail: [lidl@szu.edu.cn](mailto:lidl@szu.edu.cn) (Dr. Delong Li)

[^#^] These authors contribute equally to this study and share the first authorship.


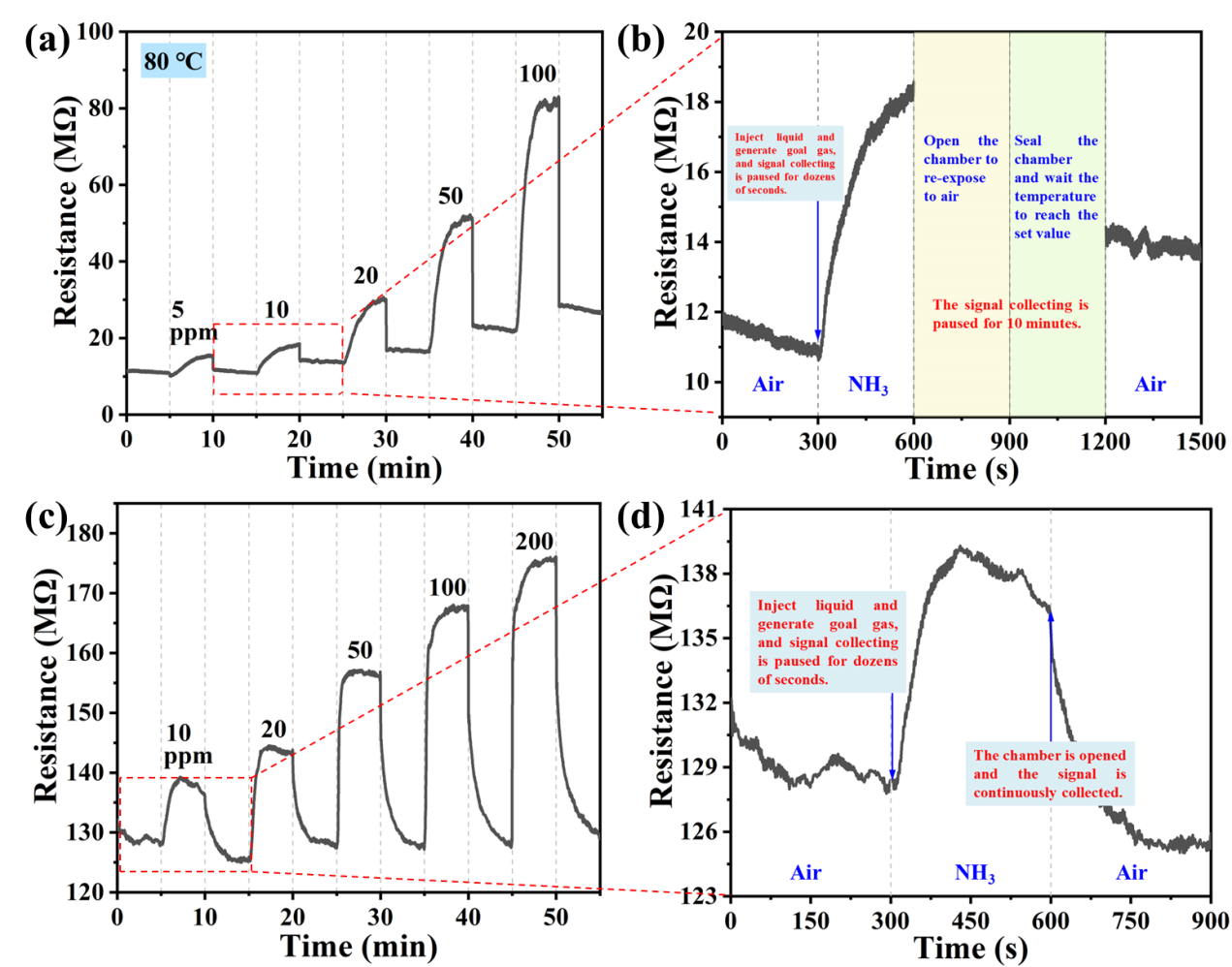


**Fig. S1** (a) Resistance variation to ammonia gas and (b) process to test the response and recovery properties to 10 ppm ammonia of BG-based sensor at 80 ℃; (c) resistance variation of ammonia gas and (d) process to test the response and recovery properties to 10 ppm ammonia of BG/Ti_3_CN (1:1) sensor at room temperature.

**Fig. S1** shows process to test the gas sensing response and recovery. In detail, during the sensing measurement, sensor device was firstly exposed to air atmosphere and sensing signals were simultaneously collected. Next, the signal collecting was paused for a short period, during which a certain amount of liquid, such as aqueous ammonia, methanol, benzene, acetone and *n-*hexane, was injected into the chamber and was immediately evaporated to generate a specific concentration of testing gas. Once the liquid was completely evaporated, signal was immediately collected again for another 5 min. Then, to characterize recovery property, the sensor device was exposed to air again by just opening the test chamber.

There was difference in characterization of recovery properties for pure BG framework and BG/Ti_3_CN composite. For the BG-based sensor operating above room temperature, as shown in **Fig. S1a** and **S1b**, when the test chamber was open to re-expose the sensor to air, airflow would have influences on the temperature of the sensor device and thus the resistance of sensing material. Hence, for the pure BG, the signal collecting was paused during the open state of the chamber. After the chamber was sealed again, another 5 min was demanded to stabilize temperature and gas flow. For the composite-based sensor operating at room temperature, the situation was much simpler. The signal was continuously collected during the recovery process even when the chamber was open to re-expose the sensor to air.


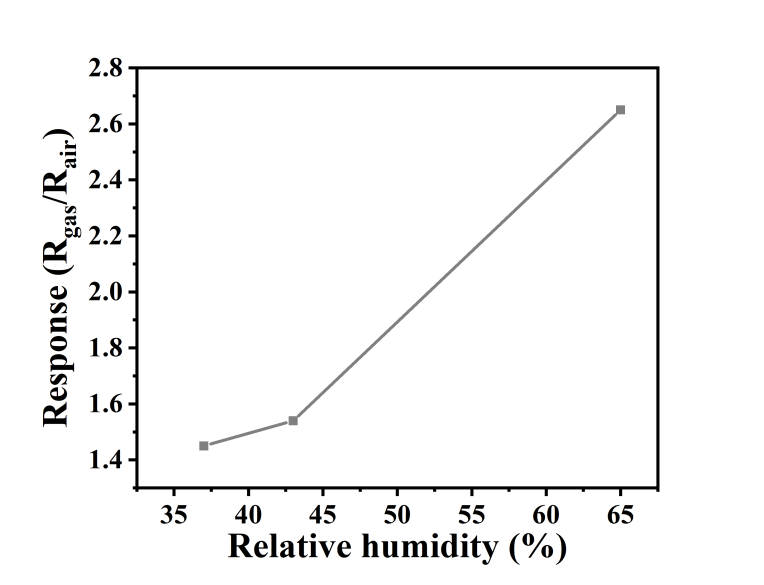


**Fig. S2** Correlation between response and humidity for pure BG to 20 ppm ammonia at 80 ℃


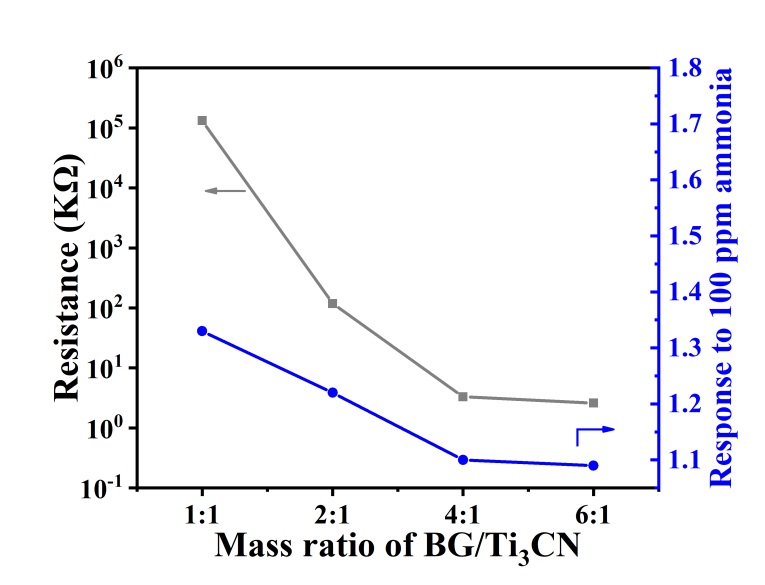


**Fig. S3** Variation of resistance and gas response to 100 ppm ammonia of BG/Ti_3_CN mixture by different ratio.


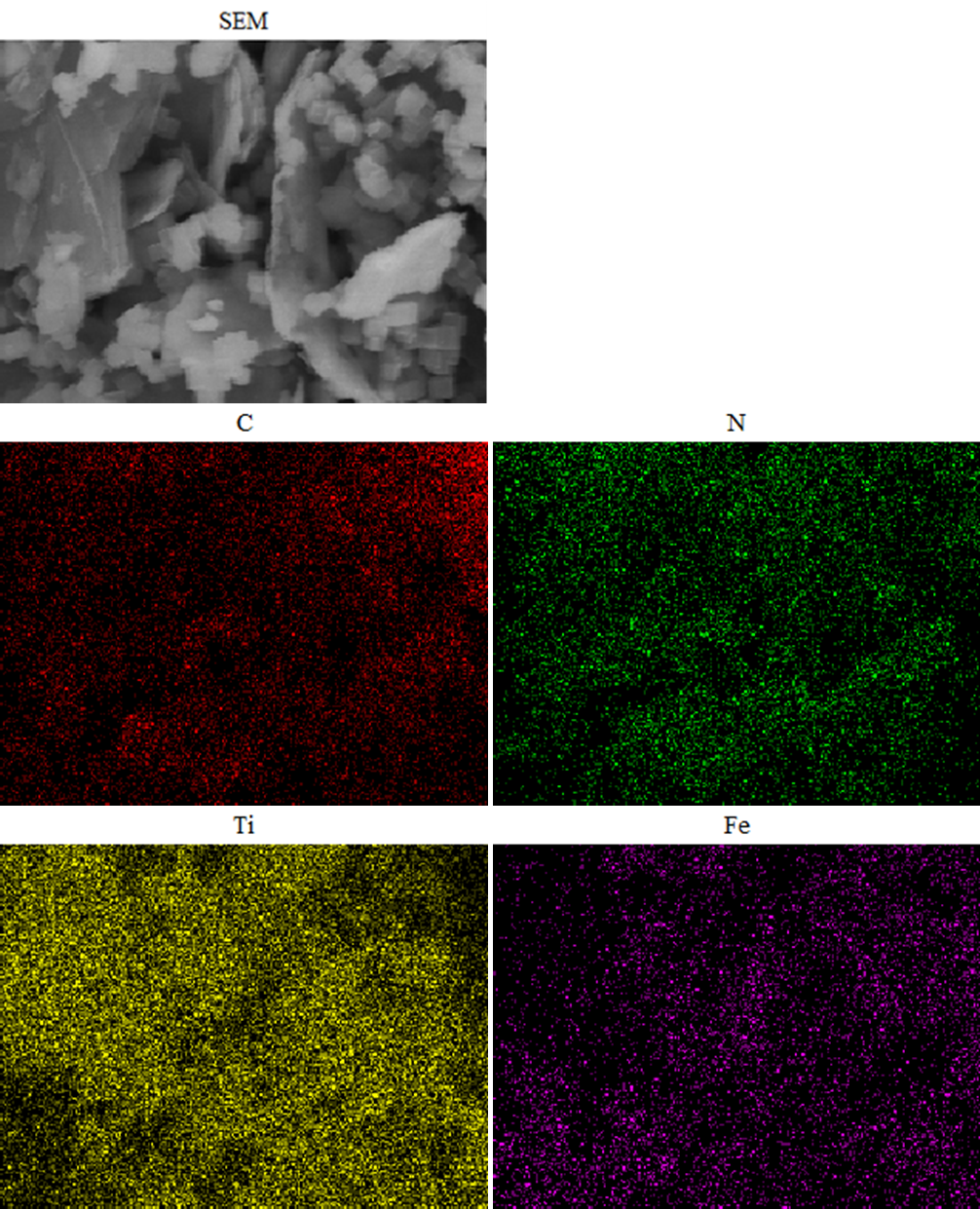


**Fig. S4** EDS mapping of BG/Ti_3_CN (1:1).

**Fig. S4** shows EDS mapping of BG/Ti_3_CN (1:1), and it is clear that C, N, Ti, Fe elements are evenly distributed so that BG and Ti_3_CN are well mixed.


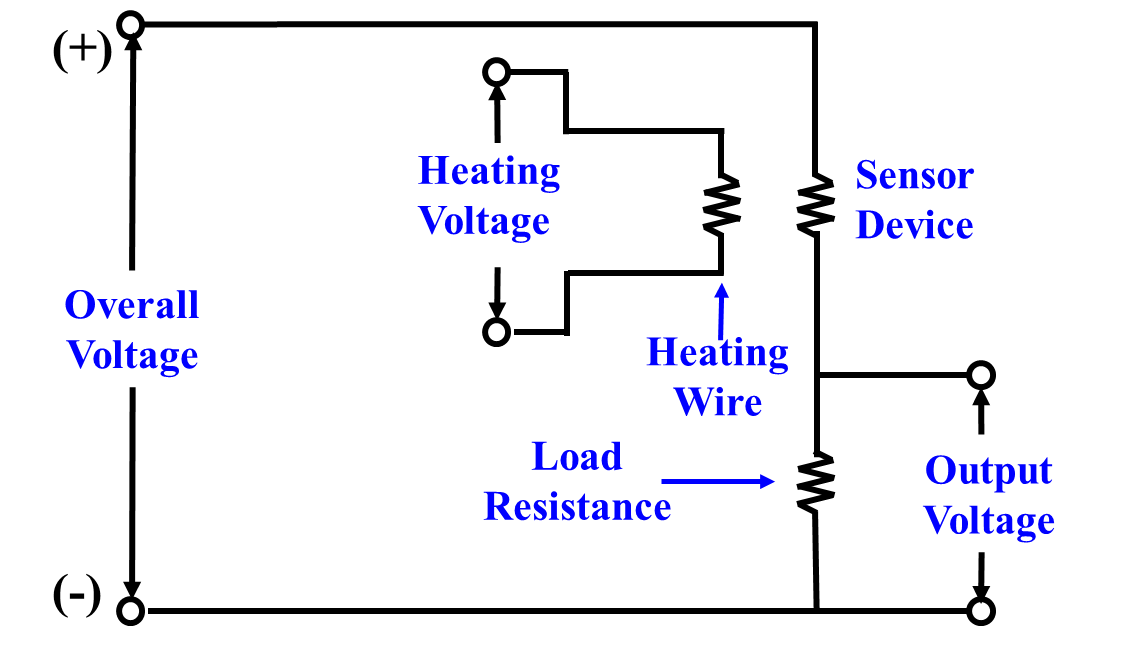


**Scheme S1** schematic diagram of WS-30A gas sensing measurement system
